# Cognitive caching promotes flexibility in task switching: evidence from event-related potentials

**DOI:** 10.1038/srep17502

**Published:** 2015-12-08

**Authors:** Florian Lange, Caroline Seer, Dorothea Müller, Bruno Kopp

**Affiliations:** 1 Department of Neurology, Hannover Medical School, Hannover, Germany

## Abstract

Time-consuming processes of task-set reconfiguration have been shown to contribute to the costs of switching between cognitive tasks. We describe and probe a novel mechanism serving to reduce the costs of task-set reconfiguration. We propose that when individuals are uncertain about the currently valid task, one task set is activated for execution while other task sets are maintained at a pre-active state in cognitive cache. We tested this idea by assessing an event-related potential (ERP) index of task-set reconfiguration in a three-rule task-switching paradigm involving varying degrees of task uncertainty. In high-uncertainty conditions, two viable tasks were equally likely to be correct whereas in low-uncertainty conditions, one task was more likely than the other. ERP and performance measures indicated substantial costs of task-set reconfiguration when participants were required to switch away from a task that had been likely to be correct. In contrast, task-set-reconfiguration costs were markedly reduced when the previous task set was chosen under high task uncertainty. These results suggest that cognitive caching of alternative task sets adds to human cognitive flexibility under high task uncertainty.

Variable and uncertain environments pose an immense challenge on the flexibility of human cognition in everyday life. How do our minds and brains manage to keep up with environmental change and to shift between different cognitive tasks on a frequent basis? Research employing the experimental task-switching paradigm has provided some intriguing answers to this question by advancing our understanding of the mechanisms underlying and the limitations constraining cognitive flexibility^1^,^2^. When individuals are required to switch tasks, it is now widely accepted that time-consuming processes of active task-set reconfiguration (TSR) are recruited to allow for successful adaptation^3^. TSR is thought to involve the retrieval of an inactive task set from long-term memory into procedural working memory (WM)^2^,^4^. This notion does not necessarily imply that task-set activation occurs in an all-or-none fashion. In fact, influential task-switching models assume that all viable task sets are represented simultaneously while only quantitative differences in attentional weights determine which task will be executed^5^,^6^. It is unlikely that processing resources focus entirely on the relevant task set while the irrelevant task set is completely deactivated. Here, we build on the idea that task sets can not only be represented in an active or an inactive state, but also in a preactive state^7^. This third activation state may provide human cognition with an additional degree of flexibility. Specifically, we propose that when it is not clear which of two alternative tasks is most adaptive in a given situation, one task is retrieved for execution into WM, while the other task is maintained in a *cognitive cache*. When incoming information then favors a shift to the task set in cache, information relevant to the new task can be retrieved fast and efficiently.

This notion is especially appealing because a mechanism of cognitive caching would enable the concurrent pursuit of two different task goals^7^. The existence of a system simulating an alternative to overt action would raise human cognitive flexibility to a new level^7^. Drawing on a cognitive cache, human cognition would not only be adaptive, but preadaptive (i.e., able to adjust to changes that are sufficiently likely to occur in the future)^8^.

Our model of cognitive caching is closely related to notions of counterfactual thinking^9^, mental simulation^10^ or what Jonathan Evans has called hypothetical thinking^11^. According to Evans^11^, the ability to mentally simulate future possibilities is characteristic for the distinctively human functions of the reflective mind. Consistent with this idea, fMRI studies have mapped the cognitive cache onto the frontopolar cortex^7^,^12^,^13^, a cortical region that has evolved late in phylogeny and that has no equivalent in the brain of non-human primates^14^.

Constrained by these neuroimaging data, our model conceives the involvement of a cognitive cache to be dependent on the uncertainty over task sets in a given situation. Cognitive caching occurs selectively when subjects are uncertain about which of two alternative tasks is currently prevailing (i.e., when it would be “too risky to simply abandon one”^7^ (p. 595)). In other words, information relevant to an alternative task should only be cached when the task is sufficiently likely to be valid (see Fig. 1).

The aim of the present analysis was twofold: First, we aimed at producing initial evidence for cognitive caching in the context of task switching and at examining whether this mechanism has the potential to promote cognitive flexibility. Second, we addressed the question whether recruitment of a cognitive cache is indeed sensitive to the probabilistic context, i.e., to the current level of task uncertainty.

Participants’ performance was examined when switching tasks under high-uncertainty (two tasks are equally likely) and low-uncertainty (one task is more likely than the other) conditions (Fig. 1). We hypothesized that in low-uncertainty conditions, participants largely refrain from cognitive caching. When the initially chosen task proves to be invalid, information regarding the alternative task cannot be retrieved from cache, rendering TSR slow and inefficient. In high-uncertainty conditions, however, retrieval of the alternative task set should be less costly as it is kept at a pre-active representation level in cognitive cache.

Our analysis of behavioral performance was complemented by the investigation of switch-specific event-related potentials (ERPs). Task-switching is reliably associated with a prominent, positive-going waveform over parietal electrodes^15^,^16^,^17^,^18^,^19^,^20^. This *posterior switch positivity* (PSP) is commonly interpreted as a direct measure of the neural substrates underlying anticipatory TSR^21^. Being proportional to the neural costs of TSR, the amplitude of the PSP was expected to be lower when the alternative task set can be retrieved from cognitive cache (i.e., in high-uncertainty conditions). Similarly, in these conditions, response times and errors rates were predicted to be reduced when compared to low-uncertainty conditions without cache involvement.

## Methods

### Participants

Eighteen young and healthy students (14 female; one left-handed; mean age = 25.6 years, SD = 6.7 years, range 19–42 years) participated for course credit. All participants had normal or corrected-to-normal vision. In line with previous task-switching experiments using the ERP technique, we initially aimed at a sample size of 24^16^,^22^. Unfortunately, we had to exclude the first six participants because their EEG data could not be analyzed due to technical problems during the recording session. The study has been approved by the Ethics Committee of Hannover Medical School and was carried out in accordance with the approved guidelines. Informed consent was obtained from all subjects.

### Task and Apparatus

Participants completed variants of a three-rule task-switching procedure modeled after the Wisconsin Card Sorting Test (WCST)^23^,^24^,^25^. The task was designed using Presentation® software (Neurobehavioral Systems, Albany, CA) and displayed on a 24 inch flat screen (Eizo EV2416W, Eizo, Hakusan, Japan). Responses were collected by a Cedrus response pad (RB 830, Cedrus, San Pedro, CA).

**Figure 2:**
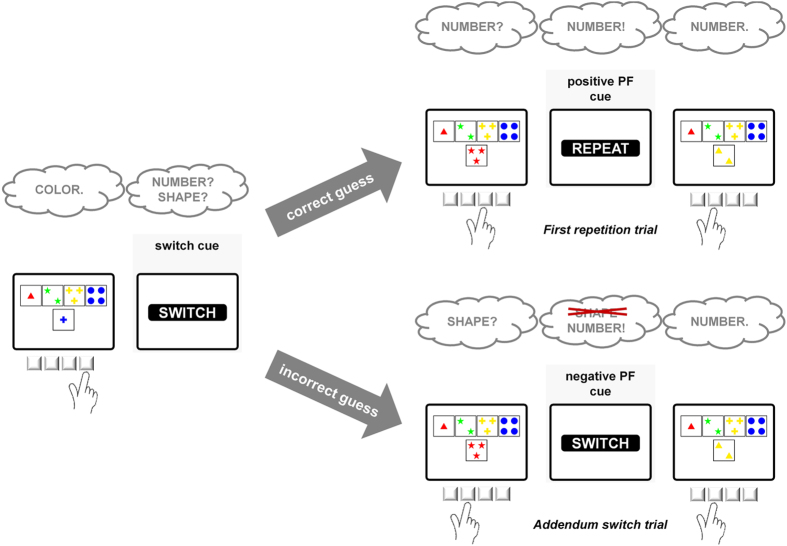
Task flow of the task-switching paradigm modeled after the Wisconsin Card Sorting Test. Participants were instructed to match a target card (by pressing a respective key) to one of four key cards according to the correct task. Feedback cues following each sorting response indicated whether the applied task had to be switched or repeated. Following a switch cue, participants were required to guess which of the two remaining tasks was now correct. If they had guessed correctly, they were presented with positive post-switch feedback (positive PF) initiating a first repetition trial. If they had guessed incorrectly, they were presented with negative post-switch feedback (negative PF) initiating an addendum switch trial. Both positive and negative PF allowed inducing the correct task rule, but only negative PF required an additional switch of tasks.

The task switching paradigm required participants to match a target card to one of four key cards according to a particular sorting rule that changed from time to time^17^,^26^,^27^. Cards were configured around the center of the screen with the target card appearing below the key cards (see Fig. 2). Participants indicated their sorting choice by pressing one of four keys on the response pad which mapped to the spatial position of the key cards on the screen. Target cards varied on three dimensions (color, shape, number), and these dimensions equaled the three viable task rules. As the target card never shared more than one attribute with any of the keycards, the applied sorting rule could unambiguously be identified^28^,^29^. Target displays remained on screen until a response was registered.

After a fixed response-cue interval (RCI) of 800 ms, a feedback cue was presented for 400 ms indicating whether the applied sorting rule should be maintained (“REPEAT”, “STAY”) or changed (“SWITCH”) on the upcoming trial. Feedback cues were presented in white letters (31 point Arial) in a black box in the center of the screen. Subsequent target stimuli appeared after a fixed cue-target interval (CTI) of 1200 ms.

We used two different repeat cues to avoid confounding of task-switch costs and cue-switch costs^30^. Because the comparisons presented here are not affected by this confound, this methodological detail will not be of further relevance. As a side note, no significant main effect or interaction involving cue sequence could be detected on any of our measures.

The task switching paradigm we used resembled the search task introduced by Mayr and Keele with one critical exception^31^. In their paradigm, a task cue explicitly indicated which of three possible stimulus dimensions is currently relevant. In contrast, we used transition cues^32^ that provided only implicit information about the currently valid task rule in order to be able to study the role of task uncertainty in task switching. Specifically, switch cues (i.e., “SWITCH” feedback after a series of rule repetitions) signaled that the previously adopted task rule had changed, thus rendering participants uncertain about which one of the two remaining rules was correct^33^. Ideally performing participants would choose one of the two remaining rules on the subsequent trial (i.e., the *switch trial*). In the remainder of this article, the feedback cues that followed participants’ initial switching attempt are called *post-switch feedback* (PF) cues. PF could either be positive (positive PF) or negative (negative PF). While positive PF signaled that participants had chosen the correct rule which now can be maintained, negative PF indicated that participants had to switch rules again in order to find the correct new rule. We refer to the trials characterized by this demand for a further switch of sorting rules (i.e., the trials following negative PF) as *addendum switch trials*. In contrast, trials following positive PF are called *first repetition trials*.

### Design and Procedure

Rules changed in an unpredictable manner^34^ after runs of two or more repetition trials. Switch probability was manipulated by varying the mean length of task runs across blocks (high switch probability: *M* = three repetition trials; low switch probability: *M* = four repetition trials). Each participant completed three blocks of trials amounting to 112 task runs in both the high switch probability condition and the low switch probability condition. The order of high switch probability blocks and low switch probability blocks was counterbalanced across participants. Switch probability was manipulated to evaluate whether PSP amplitude simply varied as a function of event probability (see Discussion).

More crucially, we manipulated the conditional probability of one of the two remaining rules being correct when participants switched away from the third rule. In high-uncertainty conditions, both possible rules were equally likely to be correct. In low-uncertainty conditions, one of the possible rules was correct in 70% of the cases. By developing a pseudorandomized rule sequence, we ascertained that the actual frequencies of transitions from one rule to another corresponded to these conditional probabilities in each participant and in each of the switch probability conditions.

As a consequence of this manipulation, one of the three rules was more frequent than the other two. Participants were explicitly instructed about the most frequent rule and about the conditional probabilities for the rule transitions. The more frequent rule (color, shape, or number) was counterbalanced across participants.

Prior to each of the switch probability conditions, eight practice runs were administered. Participants were informed about the three possible sorting criteria and about the fact that the valid task rule would change from time to time in an unpredictable manner. They were told to attend to the feedback cues in order to infer the correct task rule.

### Electrophysiological Recordings

Continuous electroencephalogram was recorded from 30 active Ag-AgCl electrodes (BrainProducts, Gilching, Germany) placed according to the international 10–20 system. BrainVision Recorder software (Brain Products, Gilching, Germany) was used. Electrode impedance was kept below 10 kΩ. Electrodes were referenced to FCz electrode. Vertical (vEOG) and horizontal (hEOG) electrooculogram were recorded with two additional electrodes positioned at the suborbital ridge and the external ocular canthus of the right eye. EEG and EOG channels were digitized at 250 Hz and amplified using a BrainAmp amplifier (Brain Products, Gilching, Germany).

### Data Analysis

Statistical analyses were performed using SPSS 22.0 (IBM, Armonk, NY). The level of significance was set at α = 0.01. Effect size confidence intervals were calculated according to the method proposed by Steiger^35^ using the syntax developed by Karl Wuensch (core.ecu.edu/psyc/wuenschk/SPSS/CI-R2-SPSS.zip).

### Behavioral Data

Mean response times (RTs) and error rates (ER) were calculated separately for switch trials, addendum switch trials, and first repetition trials. For RT analysis, we excluded RTs faster than 100 ms or slower than 3000 ms. Trials were only included when participants sorted according to a viable rule. Hence, switch trials were only included when participants switched to one of the two remaining rules (i.e., when they did not perseverate). Addendum switch trials were only considered when participants did not perseverate on the previous switch trial and when they switched to the correct rule on the addendum switch trial. First repetition trials were included when participants repeated sorting according to the correct rule. For ER analysis, trials were considered incorrect when participants did not sort according to a viable rule. Hence, responses on switch trials were only considered incorrect when participants committed a perseverative error. Responses on addendum switch trials (i.e., when participants were given the possibility to know the identity of the correct rule) were considered incorrect when participants did not identify the correct task. Addendum switch trials following a perseverative error on the switch trial were excluded from ER analysis because on these trials participants did not receive the information necessary to infer the correct rule. Responses on first repetition trials were considered incorrect when participants did not maintain the previously applied rule.

Effects of task uncertainty and switch probability on switching performance were evaluated by means of 2 (task uncertainty: high uncertainty vs. low uncertainty) × 2 (switch probability: high probability vs. low probability) × 2 (trial type: switch vs. first repetition) analyses of variance (ANOVAs) on RT and ER. Effects of task uncertainty and switch probability on addendum switching were evaluated by means of 2 (task uncertainty: high uncertainty vs. low uncertainty) × 2 (switch probability: high probability vs. low probability) × 2 (trial type: addendum switch vs. first repetition) analyses of variance (ANOVAs) on RT and ER.

### Electrophysiological data

Electrophysiological data were evaluated using BrainVision Analyzer 2.0 (Brain Products, Gilching, Germany). After filtering (high-pass: 0.5 Hz, low-pass: 70 Hz, notch: 50 Hz), data were screened for non-stereotyped artifacts and subjected to an ocular-correction independent component analysis^36^ for further removal of ocular, muscular, and cardiac artifacts. Data were re-referenced to a common average reference offline, and segmented into epochs from −200 to 1000 ms relative to the onset of feedback cues. After baseline-correction (−200 to 0 ms), residual artifacts were rejected semi-automatically before data were averaged.

ERPs were locked to switch cues, positive PF cues and negative PF cues. PSP was analyzed in the time window from 600 to 800 ms at electrode Pz. PSP was defined as the switch-related amplitude increase of the sustained positive potential typically observed at this time point and location^37^,^17^. Effects of task uncertainty and switch probability on switch-related amplitude modulations were evaluated by means of a 2 (task uncertainty: high uncertainty vs. low uncertainty) × 2 (switch probability: high probability vs. low probability) × cue type (switch vs. positive PF) ANOVA. Effects of task uncertainty and switch probability on addendum switch-related amplitude modulations were evaluated by means of a 2 (task uncertainty: high uncertainty vs. low uncertainty) × 2 (switch probability: high probability vs. low probability) × cue type (negative PF vs. positive PF) ANOVA. Note that this latter ANOVA was performed to evaluate the key hypothesis of our study, namely that the neural costs of reconfiguration (as indexed by PSP amplitude) are lower when the alternative task set has been cached (i.e., in high-uncertainty conditions) before negative PF signals the need for a further task switch on addendum switch trials.

## Results

An overview of switch-specific and addendum-switch-specific statistical results including effect sizes and confidence intervals is given in Table 1. In the following, effects will be described in more detail when their 98% confidence intervals do not include zero (corresponding to an α level of 0.01)^35^.

### Switch-specific effects

**Figure 3:**
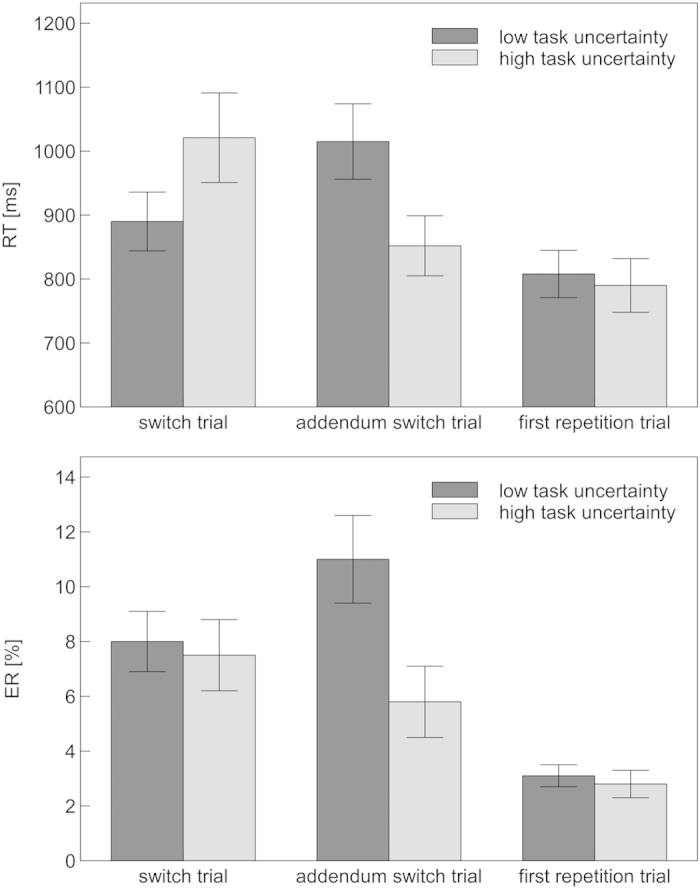
Mean latency and accuracy of responses on switch, addendum switch and first repetition trials. Error bars indicate standard error of the mean.

The task uncertainty × switch probability × trial type ANOVA revealed a significant main effect of trial type on mean RT, *F*(1, 17) = 31.01, *p *< 0.001, η_*p*_^2^ = 0.65. Responses were substantially slower on switch (955 ms) than on first repetition trials (799 ms). This effect was qualified by a significant task uncertainty × trial type interaction, *F*(1, 17) = 14.86, *p* = 0.001, η_*p*_^2^ = 0.47, indicating that RT switch costs were lower in the low-uncertainty condition (82 ms) than in the high-uncertainty condition (230 ms; see Fig. 3).

With regard to ER, the task uncertainty × switch probability × trial type ANOVA showed an effect of trial type, *F*(1, 17) = 20.14, *p* <0.001, η_*p*_^2^ = 0.54, with more errors on switch (8%) than on first repetition trials (3%).

**Figure 4:**
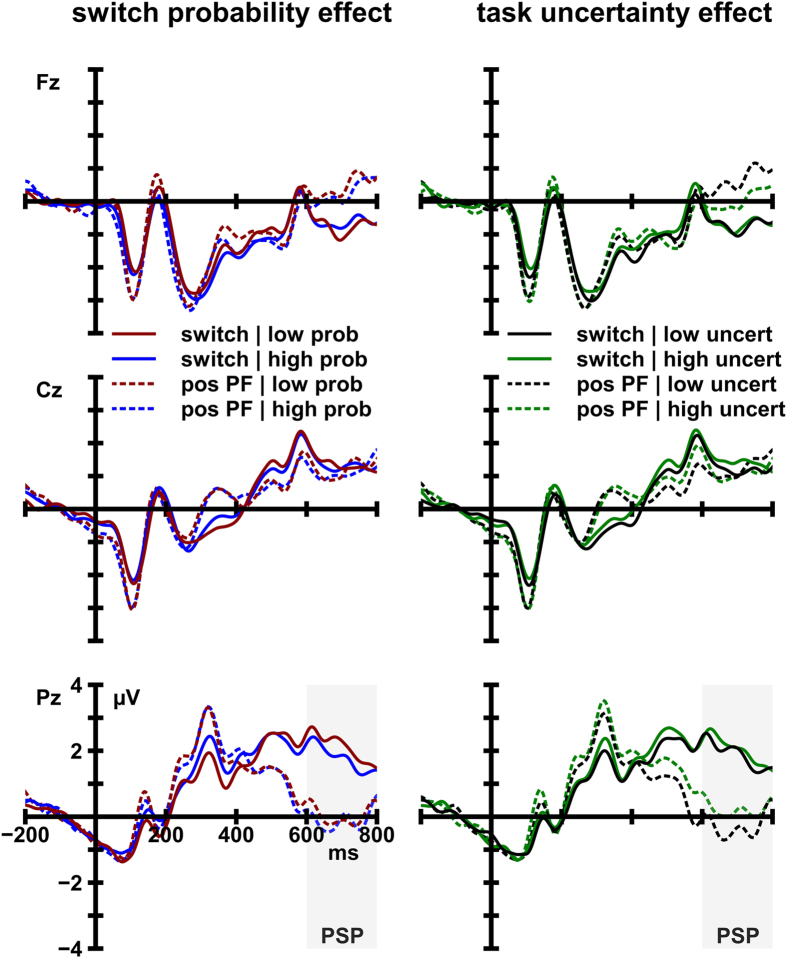
Grand average ERP waves at midline sites elicited by switch cues and positive post-switch feedback (pos PF) cues. Waveforms are depicted as a function of switch probability (low probability [low prob] vs. high probability [high prob] (left) and task uncertainty (low task uncertainty [low uncert] vs. high task uncertainty [high uncert] (right).

The analysis of PSP amplitude at electrode Pz yielded a significant main effect of cue type, *F*(1, 17) = 24.65, *p* < 0.001, η_*p*_^2^ = 0.59, in the absence of any significant effects or interactions involving the factors task uncertainty and switch probability. The sustained positive potential was larger following switch cues (1.97 μV) than after positive PF cues (−0.03 μV). This effect is displayed in Fig. 4. Inspection of Fig. 4 also reveals that PSP amplitude is neither sensitive to the probability of a switching event nor to the degree of task uncertainty per se.

### Addendum-switch-specific effects

The task uncertainty × switch probability × trial type ANOVA revealed significant main effects of trial type, *F*(1, 17) = 35.30, *p* < 0.001, η_*p*_^2^ = 0.68, and task uncertainty, *F*(1, 17) = 12.04, *p* = 0.003, η_*p*_^2^ = 0.42, on mean RT. Responses were substantially slower on addendum switch (934 ms) as compared to first repetition (799 ms) trials and in low-uncertainty (912 ms) as compared to high-uncertainty conditions (821 ms). These effects were moderated by a significant task uncertainty × trial type interaction, *F*(1, 17) = 15.64, *p* = 0.001, η_*p*_^2^ = 0.48, indicating that RT addendum switch costs were higher in the low-uncertainty condition (207 ms) than in the high-uncertainty condition (62 ms; see Fig. 3).

The same pattern could be observed with regard to ER, with significant main effects of trial type, *F*(1, 17) = 15.64, *p* = 0.001, η_*p*_^2^ = 0.48, and task uncertainty, *F*(1, 17) = 21.31, *p* < 0.001, η_*p*_^2^ = 0.56, as well as a task uncertainty × trial type interaction, *F*(1, 17) = 11.00, *p* = 0.004, η_*p*_^2^ = 0.39. Participants committed more errors on addendum switch (8%) than on first repetition trials (3%) and in low-uncertainty (7%) as compared to high-uncertainty conditions (4%). ER switch costs were larger in low-uncertainty (8%) as compared to high-uncertainty conditions (3%).

**Figure 5:**
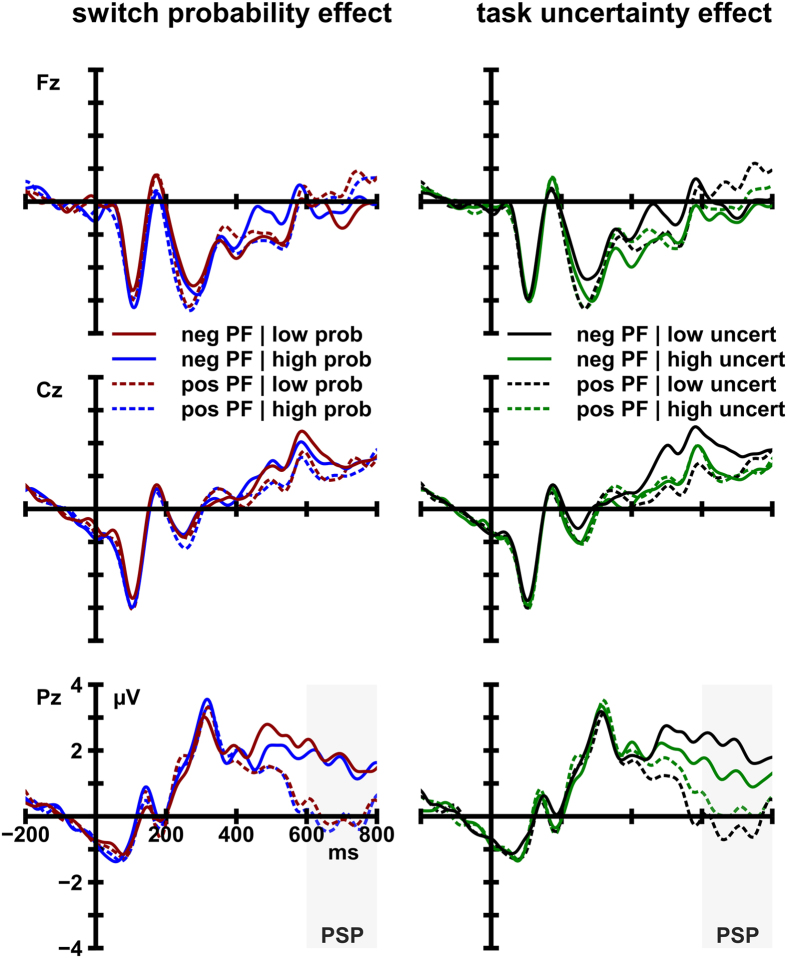
Grand average ERP waves at midline sites elicited by negative post-switch feedback (neg PF) cues and positive post-switch feedback (pos PF) cues. Waveforms are depicted as a function of switch probability (low probability [low prob] vs. high probability [high prob]) (left) and task uncertainty (low task uncertainty [low uncert] vs. high task uncertainty [high uncert]) (right).

**Figure 6:**
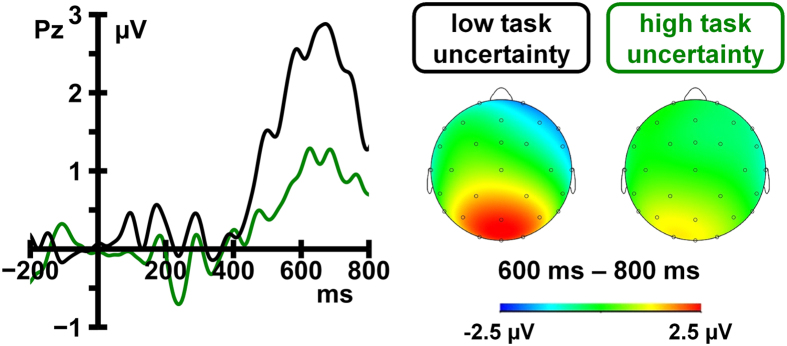
Difference waves and scalp maps illustrating the effect of task uncertainty on neural addendum switch costs. ERP waveforms elicited by positive post-switch feedback cues were subtracted from ERP waveforms elicited by negative post-switch feedback cues to obtain the neural activity specific to addendum switch operations (left). Scalp maps (right) depict the topography of this addendum-switch-specific activity.

Similar to the switch-cost analysis, the amplitude of the sustained positive potential was significantly larger following negative PF (1.67 μV) than after positive PF cues (-0.03 μV), *F*(1, 17) = 30.12, *p* < 0.001, η_*p*_^2^ =0.64. Hence, a PSP could also be observed on addendum switch trials. Importantly, the amplitude of the PSP was substantially larger in low-uncertainty conditions (2.34 μV) as compared to high-uncertainty conditions (1.04 μV). A significant interaction of the factors cue type and task uncertainty, *F*(1, 17) = 15.23, *p* = 0.001, η_*p*_^2^ = 0.41, corroborates that PSP amplitude was reliably modulated by task uncertainty (Figs 5 and 6).

## Discussion

Using variants of a task-switching paradigm, we investigated the behavioral and neural costs of switching to a new task as a function of task uncertainty. Switching was faster and more efficient when uncertainty about the correct task was low. However, when the chosen task proved to be invalid (i.e., when participants had to switch again on addendum switch trials) the costs of switching to an alternative task were substantially increased in these low-uncertainty conditions. This pattern of increased addendum switch costs in low-uncertainty conditions was not only evident in the speed and accuracy of responses to target stimuli, but also in the amplitude of the PSP, an ERP measure of anticipatory TSR prior to target onset.

These data are suggestive of the uncertainty-dependent recruitment of a cognitive cache. When participants can be relatively certain about the currently valid task, they appear to commit to this particular task: they activate the corresponding task set for execution and do not (or to a substantially lesser extent) keep information relevant to an alternative task at a pre-active representation level in cognitive cache. When performance feedback then signals that participants have committed to the wrong task, retrieving the alternative task set from its relatively inactive state in long-term memory is costly and effortful (as indicated by increased RT, ER and PSP amplitudes). These costs of TSR are remarkably reduced when the alternative task set can be retrieved from a pre-active state in cognitive cache. We propose that the cognitive cache is selectively recruited in situations involving high uncertainty about the currently valid task. In these situations, participants do not only retrieve one task set into an active state for execution, but they also prepare for the hypothetical but not unlikely event that the chosen task is not correct. Specifically, our model assumes that they retrieve the alternative task set from long-term memory into a pre-active state in cognitive cache. This process of cognitive caching, mental simulation^10^, or hypothetical thinking^11^ appears to substantially promote participants’ flexibility in the task-switching procedure: the alternative task set in cognitive cache is more readily accessible and, as a consequence, the behavioral and neural costs of TSR on addendum switch trials are lower in high-uncertainty conditions.

**Figure 7:**
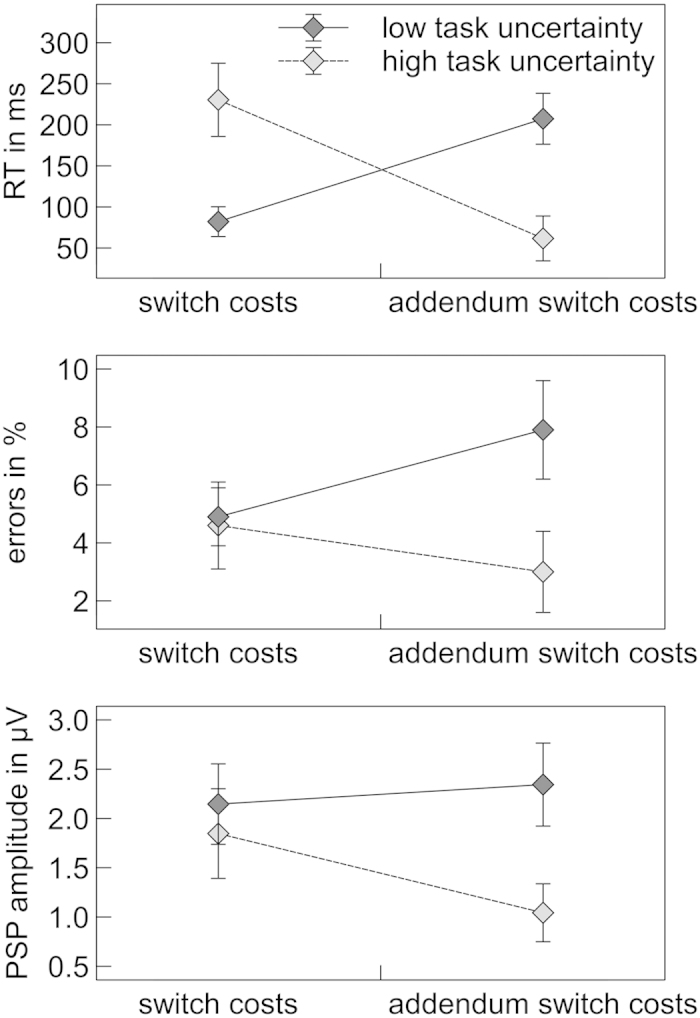
Behavioral and electrophysiological switch costs (switch trial – first repetition trial) and addendum switch costs (addendum switch trial – first repetition trial), separately for low-uncertainty conditions and high-uncertainty conditions. Error bars indicate standard error of the mean.

Our methodological approach of combining behavioral and electrophysiological measures and distinguishing between switch and addendum switch conditions allowed us to rule out some alternative explanations for the observed pattern of results. First, it might be argued that increased addendum switch costs in low-uncertainty conditions are not due to an increased demand for TSR but rather to stronger effects of task-set inertia^38^. When participants have to switch away from a task they have committed to, the activation of the associated task set and, hence, the interference with the response to the next target stimulus might be stronger. However, such a stronger tendency to sort a target stimulus according to the old task set would only explain effects of task uncertainty on *behavioral* addendum switch costs. The effect of task uncertainty on PSP amplitude was observed *prior* to target onset and can thus not be attributed to the interference of task-set inertia with processing of the target stimulus. Future studies manipulating the CTI and the RCI might further differentiate the processes contributing to the uncertainty-related modulation of addendum switch costs observed in our study. Second, the nature of our manipulation of task uncertainty dictates that negative PF is less likely in the low-uncertainty condition than in the high-uncertainty condition. Hence, larger PSP amplitudes following negative PF in the low-uncertainty condition might result from a potential effect of event probability on PSP amplitude. However, when investigating PSP responses to switch cues as a function of switch probability, we did not find PSP amplitude to be probability sensitive. This result is consistent with previous studies suggesting that late (>500 ms) PSP waveforms reflect TSR processes that are not affected by the switch probability context^22^,^39^. Third, it is possible that negative PF in low-uncertainty conditions render participants confused and uncertain about the currently valid task and increased PSP amplitudes might reflect processes of signaling or overcoming this uncertainty. Again, this explanation does not account for our data because PSP amplitudes on switch trials did not vary as a function of task uncertainty (Fig. 7).

In future studies, it may prove worthwhile to examine the similarities between the idea of cognitive caching and extant models of working memory processes. The models of Cowan^40^ and Oberauer^41^, for example, refer to different functional states of information in working memory. According to these models, the information used in ongoing cognitive processes is held in a region of direct access (i.e., inside the focus of attention), whereas an additional subset of long-term memory is activated outside this focus of attention. In fact, this distinction between working memory information inside and outside the focus of attention has already been linked to the relationship between active task sets and cached task sets^42^. However, further theoretical and empirical work is required to investigate commonalities between these different approaches to human cognition.

To conclude, the observed pattern of reduced addendum switch costs following task selections under high uncertainty can be best explained by the strategic recruitment of a cognitive cache. Being uncertain about which task to pursue, individuals necessarily decide for one task, but they do not completely discard alternative tasks if these are also sufficiently likely to be valid. The process of simulating and preparing the alternative appears to be highly adaptive as it allows for smooth adjustments to constantly changing environmental demands.

## Additional Information

**How to cite this article**: Lange, F. *et al.* Cognitive caching promotes flexibility in task switching: evidence from event-related potentials. *Sci. Rep.* **5**, 17502; doi: 10.1038/srep17502 (2015).

## Figures and Tables

**Figure 1:**
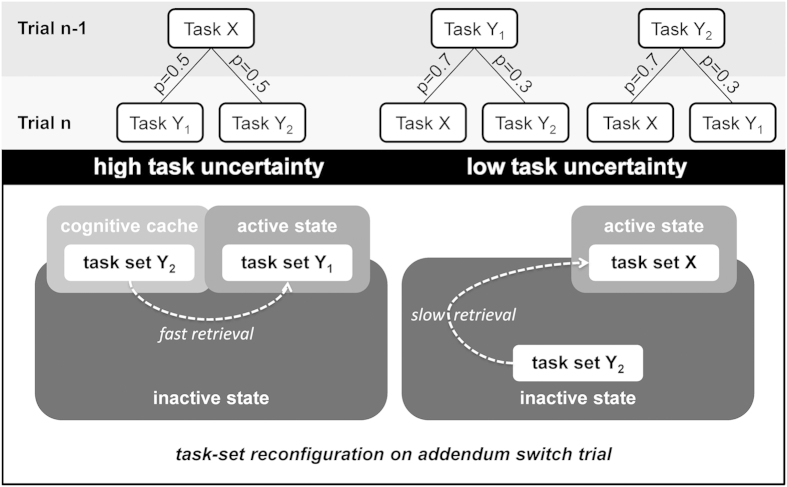
Illustration of our manipulation of task uncertainty (top) and its hypothesized effects on the activation of task sets (bottom). Under high task uncertainty, participants had to decide between two tasks that were equally likely to be correct. We propose that in these situations, individuals activate one task set for execution and keep the unchosen task set in a pre-active state in cognitive cache, thereby facilitating later retrieval of this task set. Under low task uncertainty, participants knew that one task was more likely to be correct than the other. We propose that in these situations, individuals largely refrain from cognitive caching, rendering later retrieval of the alternative task set slow and inefficient.

**Table 1:** Overview of statistical results.

Measure	Factor or interaction	*F*	*p*	ηp^2^	98% CI
Switch costs					
Response time	**trial type**	**31.01**	**<0.001**	**0.646**	**0.224–0.801**
	task uncertainty	4.15	0.058	0.196	0.000–0.513
	switch probability	0.10	0.757	0.006	0.000–0.185
	**trial type** × **task uncertainty**	**14.86**	**0.001**	**0.466**	**0.054**–**0.697**
	trial type × switch probability	0.75	0.400	0.042	0.000–0.351
	task uncertainty × switch probability	0.76	0.395	0.043	0.000–0.351
	trial type × task uncertainty × switch probability	0.46	0.506	0.026	0.000–0.322
Error rates	**trial type**	**20.14**	**<0.001**	**0.542**	**0.110**–**0.742**
	task uncertainty	0.81	0.381	0.045	0.000–0.356
	switch probability	3.19	0.092	0.158	0.000–0.481
	trial type × task uncertainty	0.04	0.854	0.002	0.000–0.083
	trial type × switch probability	0.17	0.689	0.010	0.000–0.278
	task uncertainty × switch probability	0.22	0.646	0.013	0.000–0.288
	trial type × task uncertainty × switch probability	0.00	0.959	0.000	0.000–0.000
PSP amplitude	**trial type**	**24.65**	**<0.001**	**0.592**	**0.159**–**0.770**
	task uncertainty	5.03	0.039	0.228	0.000–0.538
	switch probability	0.88	0.362	0.049	0.000–0.362
	trial type × task uncertainty	0.89	0.360	0.050	0.000–0.362
	trial type × switch probability	0.21	0.651	0.012	0.000–0.286
	task uncertainty × switch probability	0.30	0.593	0.017	0.000–0.301
	trial type × task uncertainty × switch probability	0.00	0.999	0.000	0.000–0.000
Addendum switch costs					
Response time	**trial type**	**35.30**	**<0.001**	**0.675**	**0.264**–**0.818**
	**task uncertainty**	**12.04**	**0.003**	**0.415**	**0.038**–**0.665**
	switch probability	0.00	0.985	0.000	0.000–0.000
	**trial type** × **task uncertainty**	**15.64**	**0.001**	**0.479**	**0.062**–**0.704**
	trial type × switch probability	0.01	0.908	0.001	0.000–0.022
	task uncertainty × switch probability	0.28	0.602	0.016	0.000–0.298
	trial type × task uncertainty × switch probability	4.12	0.058	0.195	0.000–0.512
Error rates	**trial type**	**15.64**	**0.001**	**0.479**	**0.062**–**0.704**
	**task uncertainty**	**21.31**	**<0.001**	**0.556**	**0.123**–**0.750**
	switch probability	0.65	0.431	0.037	0.000–0.342
	**trial type** × **task uncertainty**	**11.00**	**0.004**	**0.393**	**0.019**–**0.651**
	trial type × switch probability	0.24	0.632	0.014	0.000–0.292
	task uncertainty × switch probability	0.29	0.595	0.017	0.000–0.300
	trial type × task uncertainty × switch probability	0.57	0.461	0.032	0.000–0.334
PSP amplitude	**trial type**	**30.12**	**< 0.001**	**0.639**	**0.215**–**0.797**
	task uncertainty	0.62	0.440	0.035	0.000–0.339
	switch probability	0.14	0.712	0.008	0.000–0.241
	**trial type** × **task uncertainty**	**11.65**	**0.003**	**0.407**	**0.034**–**0.660**
	trial type × switch probability	0.01	0.921	0.001	0.000–0.022
	task uncertainty × switch probability	1.12	0.305	0.062	0.000–0.379
	trial type × task uncertainty × switch probability	0.77	0.394	0.043	0.000–0.353
